# Doctors as Policemen

**Published:** 1889-08-10

**Authors:** 


### The Hospital

A Weekly Institutional Journal of

**Science, Medicine, Nursing, and Philanthropy.**

*For Hospitals, Asylums, Medical Practitioners, Students, Nurses, Families, Parishes, Congregations, and the Charitable Public.*

Vol. VI. No. 150.] [August 10, 1889.

### Doctors as Policemen.

Few experienced persons will doubt the desirability of ensuring the compulsory notification of infectious diseases. An infectious disease, such as smallpox, scarlet fever, diphtheria, is in every sense of the word an alarming public danger, and ought to be dealt with exactly as a fire is dealt with—that is to say, it should be stamped out as quickly as possible. But whilst all are agreed as to the necessity of the stamping-out process, there is no unanimity of opinion as to the persons who are to perform it. On the contrary, there seems to be great confusion of ideas on the subject, and in some minds a complete absence of even an elementary knowledge of general principles.

The Bill moved by Mr. Ritchie last week, and which was read a second time without a division, is intended to provide for the compulsory notification of infectious diseases, and may, when passed, apply to the whole United Kingdom. The agreement in the House of Commons probably indicates a similar agreement in the country as to the great desirability of compulsory notification in itself. The debate, however, clearly showed that there was no such universal agreement as to the method to be employed. Sir Walter Foster, on one hand, expressed the decided conviction that medical men should on no account be charged with the duty and responsibility of notifying cases of infectious disease which come to their knowledge in the ordinary course of their professional avocations. Mr. Long, on the other hand, seemed to consider Sir Walter Foster's views of small moment, and argued that so long as notification was desirable in itself, the House of Commons and the country ought to hesitate a long time before they allowed any regard for the medical difficulty to interfere with necessary precautions in defence of the public health. The opinions thus set forth on both sides of the question no doubt largely prevail throughout the country.

But what is it that will be required of medical men if they should be commanded under penalties to make known to the public authorities every case of infectious disease which comes under their notice? By a single stroke of the Imperial pen they will be converted at once into a body of State police. They are not asked whether or not they are willing to serve in this capacity, nor are they promised any reward for the deprivation of liberty to which they will be subjected. But if the Bill, when passed, throws upon them the necessity of notification, they will be compelled with their will, or against their will, to render definite service to the State of a compulsory, unconstitutional, and unavoidable character. Now this cannot be permitted. It has been argued that it will be disagreeable to medical men to impose upon them the duty of compulsory notification, and that it may prove, in many cases, injurious to the practice of particular individuals; and these arguments in themselves are of very great weight. But they are as nothing compared with the dangerous infraction of liberty which it is thus proposed to make legal. On what grounds of political justice can it be shown to be right that doctors, more than any other class, should be compelled against their will to render any service to the State whatever? Many a plumber in the course of his duties finds out insanitary dangers to life in the houses of his employers and elsewhere. Who proposes that the plumber shall be compelled to notify these dangers to the public authorities? Many a solicitor in the course of his avocations is told facts of the most criminal and compromising character. Will any suggest that it is the duty of the solicitor who is called upon to defend a criminal to make known in the interests of the State all the criminatory facts that the criminal may lay bare to him? Many a priest and private friend listens to confidences which it would be advantageous for the State to have told in its private ear; but is there any person anywhere who will affirm that the priest or the private friend is bound by considerations of public duty to make known these confidences to the criminal authorities?

It is highly probable that this aspect of the question has not presented itself either to our sage legislators or to the members of the medical profession generally. Doctors in family practice have the strongest possible objections, not to compulsory notification, but to being themselves compelled to make it; and this objection no doubt is largely due to the apprehensions of injury which they entertain. The bulk of them being unaccustomed to work together, and probably feeling much more respect for the wisdom of the House of Commons than that wisdom is at all entitled to, do not quite know what to do. But if our legislators should show themselves so ignorant of the very elements of political justice and common-sense as to insist upon converting the medical profession into a body of State police, whether that profession be willing or not, then it will be the duty of all medical men, great and small, not only in their own interests, but still more in the interests of State justice, to rise up as one man and resist the measure to the utmost.

It is as plain as the sun in the heavens that there is one person and one only who ought in the last resort to be made responsible for the compulsory notification of infectious diseases which may appear in his house. That person is, of course, the parent or other individual who may stand *in loco parentis* in any given house at the time of an outbreak. That person is responsible for the payment of rent, of rates, and in fact for everything else that goes on in the house. He is the natural representative to the public authorities of all the household affairs. On what principle is it justifiable to introduce a new departure of such a fundamental and unconstitutional character as is now proposed?

What is to prevent the parent making known a case of infectious disease to the registrar exactly as he makes known a case of death? We do not make it compulsory on the doctor to notify deaths; and it would be absurd if we did. Equally absurd and equally unjust would it be to make the doctor responsible instead of the parent for the notification of an infectious disease. No doubt it is the doctor's duty to inform the parent at once when any such case arises; and he might very well be asked to explain to the parent that the duty of notification is compulsory. But beyond this there should be thrown upon the medical practitioner no direct responsibility whatever so far as the State is concerned.

If it be considered desirable or absolutely necessary in the interests of the public safety that doctors should act as State officials and notify infectious diseases, then the State must approach them exactly as through its recruiting sergeant it approaches the man whom it wishes to enlist. It must ask every medical man whether or not he is prepared to become a State official on certain definite terms. Those terms must include adequate payment for services rendered, and the fullest liberty to retire from this department of State duty at any time, and on due notice given. On these conditions, and on these alone, should doctors undertake service for the State. And we repeat that, if need be, the whole profession must stand shoulder to shoulder, and resist to the very utmost the slightest encroachment of any kind upon its personal liberties.

